# The Use of Long-term Antibiotics for Suppression of Bacterial Infections

**DOI:** 10.1093/cid/ciae302

**Published:** 2024-06-04

**Authors:** Molly Horne, Ian Woolley, Jillian S Y Lau

**Affiliations:** Faculty of Medicine, Nursing, and Health Sciences, Monash University, Clayton, Victoria, Australia; Faculty of Medicine, Nursing, and Health Sciences, Monash University, Clayton, Victoria, Australia; Monash Infectious Diseases, Monash Health, Clayton, Victoria, Australia; Faculty of Medicine, Nursing, and Health Sciences, Monash University, Clayton, Victoria, Australia; Monash Infectious Diseases, Monash Health, Clayton, Victoria, Australia

**Keywords:** antibiotics, infection, prosthetic joint infection, antimicrobial stewardship, suppression

## Abstract

Suppressive antibiotic therapy is prescribed when a patient has an infection that is presumed to be incurable by a defined course of therapy or source control. The cohort receiving suppressive antibiotic therapy is typically highly comorbid and the infections often involve retained prosthetic material. In part due to a lack of clear guidelines regarding the use of suppressive antibiotics, and in part due to the complex nature of the infections in question, patients are often prescribed suppressive antibiotics for extremely long, if not indefinite, courses. The risks of prolonged antibiotic exposure in this context are not fully characterized, but they include adverse drug effects ranging from mild to severe, the development of antibiotic-resistant organisms, and perturbations of the gastrointestinal microbiome. In this narrative review we present the available evidence for the use of suppressive antibiotic therapy in 4 common indications, examine the gaps in the current literature, and explore the known and potential risks of this therapy. We also make suggestions for improving the quality of evidence in future studies, particularly by highlighting the need for a standardized term to describe the use of long courses of antibiotics to suppress hard-to-treat infections.

## Teaser

Current guidelines for suppressive antibiotic therapy (SAT) don’t provide clear recommendations for duration and cessation of therapy, largely due to limited data in the literature. This review examines the available evidence and proposes recommendations for the major indications for SAT.

## Graphical Abstract

**Graphical Abstract:**
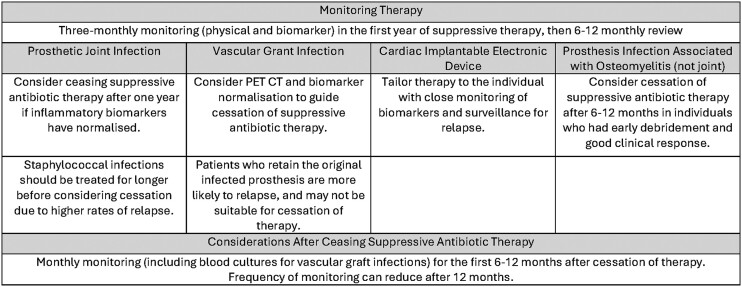

Antibiotics are a familiar therapeutic agent for clinicians, and indeed for many patients themselves. Over 211 million antibiotic prescriptions were written in the United States in 2021 [1]. This review focuses on a subsection of antibiotic use: long-term antibiotics for infections thought to be incurable. Although this represents only a small proportion of overall antibiotic use, by its nature it may represent an important opportunity for antimicrobial stewardship.

A previous study by our group found that, of 3 broad indications for long-term antibiotics, the use of antibiotics to suppress infections presumed to be incurable was supported by the least robust evidence [2]. The other broad indications of prophylactic and noninfective uses are outside the scope of this review. Long-term or indefinite antibiotics have been proposed as a treatment strategy in patients who are ill-suited to more invasive surgical methods [3]. This approach is not usually intended to cure the infection, but instead to improve symptoms and prevent progression or relapse to a point of clinical significance [4]. Our previous review identified 4 infection groups that were treated most commonly with suppressive antibiotic therapy (SAT) in a major Australian hospital network [5]—namely, periprosthetic joint infection (PJI), vascular graft infection (VGI) and other vascular infections including infective endocarditis and mycotic aneurysm, cardiac-implantable electronic device infection (CIEDI), and osteomyelitis and other orthopedic hardware–associated infections.

The guidelines for these broad infections generally recommend a combined medical and surgical approach to treatment [6–8]. In some cases, definitive source control through surgery is impossible and/or a patient may be too comorbid for surgical intervention. In such scenarios, the guidelines typically allude to SAT; however, currently, there are no specific recommendations for their use in any of the guidelines examined.

A major challenge to curing infections involving prosthetic material is the ability for many bacteria to form biofilms. Bacterial biofilms are microscopic matrix that can adhere to the surfaces of both living tissue and prosthetic hardware [9, 10] and can form rapidly following infection. Bacteria embedded in biofilms on prosthetic material can evade detection by conventional microscopy and culture [11]. The protective features of a bacterial biofilm allow it to resist antimicrobial activity, making curative efforts to treat infections involving prosthetic hardware increasingly challenging [12]. Few antibacterial agents can penetrate the biofilm; in fact, some beta-lactam antibiotics have been shown to induce the biofilm [13].

An SAT may also be used for other miscellaneous infections. However, such indications are outside the scope of this review.

## METHODS

Literature searches were conducted in November 2023. The databases searched were Ovid MEDLINE, PubMed, and Cochrane. The search strategy combined terms describing suppressive antibiotic therapy (prolonged OR long-term OR continuous OR indefinite OR suppress* AND antimicrobial OR antibiotic), with terms describing the indications described in our previous study: for PJI: PJI OR prosthe* OR joint OR arthroplasty OR replacement; for VGI: vascular OR graft OR endovascular OR endoprosthes* OR aort* OR endocar ditis OR mycotic; for CIEDI: implant* OR cardiac OR electronic OR defibril* OR ventric* OR device; for OM/other implant infection: osteomyelitis OR om OR bone OR spin* OR metalware.

Search results were limited to adult and human studies in English. Titles and abstracts were manually screened for relevance to SAT. Studies that administered long courses of antibiotics with primary curative intent were excluded.

### Periprosthetic Joint Infections

*Staphylococcus aureus* is most frequently described as the infecting organism in PJI in the literature, followed by coagulase-negative *Staphylococcus* [14–30]. Beta-lactams and tetracyclines are the most commonly prescribed antibiotics for SAT [15, 16, 18, 20, 22–27, 29, 31, 32].

Historically, SAT for PJI was associated with poor outcomes [14, 15], but success rates from the past decade have ranged between 60% and 93% [19–25, 27, 28, 31–34]. Many studies lack comparison groups, but in those that have a non-SAT control population SAT is shown to reduce the risk of infection recurrence, although not always to a level of statistical significance [29, 32, 35]. In particular, SAT used in the context of PJI with draining fistula was not found to significantly improve the risk of implant retention [30].

Several published literature reviews explore the use of SAT in PJI, often as a follow-on strategy after surgical debridement and implant retention (DAIR) [4, 27, 36, 37]. These reviews typically find favorable success rates ranging from 66% to 75%, but they acknowledge the difficulty in assessing the efficacy of SAT due to the heterogeneity of the literature at present—namely, variations between medical and surgical management prior to commencement of SAT, definitions of what therapy actually constitutes SAT, and characterization of treatment success or failure.

In the SAT literature, patients with staphylococcal infections (especially *S. aureus*) appear to have a worse prognosis than those without. Several studies have reported worse outcomes in patients with periprosthetic joint infections caused by *S. aureus* compared with those caused by other bacteria [16, 17, 21, 26, 29, 33, 38]. Although not directly comparable, other studies have found that SAT can be very effective in streptococcal PJI. A 2019 study of patients with PJI caused by streptococci found a 93% treatment success rate with SAT, compared to a 57% success rate with patients not treated with SAT (*P* = .002) [32], although an older study with very small numbers found that 50% (3/6) of patients with beta-hemolytic streptococcal infections failed treatment [39]. However, the trend of SAT patients with staphylococcal infections faring worse than those with infections caused by other organisms is not universal. Both a 2020 and 2022 study found that gram-negative infections were associated with worse SAT outcomes than gram-positive infections [28, 31]. The reason for this finding is not clear, although a review published in 2021 suggested that it may be due to the paucity of orally available antibiotics that target gram-negative organisms [4].

The ideal duration of SAT for PJI remains unclear. A 2020 study reported that the hazard rate of failing SAT did not significantly decrease beyond a treatment duration of 12 months [29]. Indeed, another study found that, while an SAT duration of less than 90 days was associated with an increased risk of failure, longer treatment durations of 90–180, 180–365, and over 365 days were not associated with any change in failure rate [19]. There is also anecdotal evidence in the literature of patients stopping SAT after an extended duration and remaining symptom free [16, 17, 26, 27, 39]. However, there are currently no definitive guidelines to determine a clinically relevant stopping point, nor a biomarker that may indicate that it is safe to cease SAT. Markers such as a normalized erythrocyte sedimentation rate (ESR) have been used to guide SAT cessation or to assess likely success of SAT as a therapeutic option [21]; however, there have been instances in the literature of patients with a normal ESR having infection recurrence between 3 days and 1 month after stopping SAT [16, 39]. Some authors have also suggested C-reactive protein (CRP) levels, serum bactericidal titers greater than 1:8, and radiolabeled leukocyte scintigraphy as possible methods for monitoring response to SAT [16, 40], but these strategies have not been extensively documented in the literature.

As previously mentioned, the heterogeneity of patient populations between, and often within, studies makes it difficult to provide recommendations for clinical practice. Acknowledging this limitation, and in light of a recent narrative review of SAT in PJI [36], we suggest the following practices when managing patients on SAT for PJI:

1. Consider SAT cessation after 1 year of continuous therapy, if the inflammatory markers, particularly ESR have normalized.
2. Patients with staphylococcal infections should be treated more conservatively, with longer duration of therapy, and a more cautious approach to ceasing SAT, due to the apparent worse prognosis of staphylococcal infections in the literature.

### Vascular Graft Infections

Most of the literature on SAT for VGI comes from case series and reports [41–49]. These case reports are published presumably because they have unique etiology, not reflective of the majority of VGIs requiring suppressive therapy. These case reports describe patients with VGI caused by organisms such as *Brucella melitensis* [43], *Edwardsiella tarda* [44], and *Capnocytophaga canimorsus* [45]. Beta-lactams are the most prescribed antibiotic agent for suppressive therapy in this context [42, 45, 50]. In several studies, the antibiotics used for long-term suppression are not specified.

The success rate of SAT for VGI reported in retrospective studies is variable, although it appears that computed tomography (CT)/positron emission tomography (PET) imaging may have utility in determining the need for ongoing SAT. A 2021 retrospective cohort study investigated the outcomes of 32 patients with infective endocarditis who did not undergo an indicated surgical procedure. In this study 24 patients received SAT for a median duration of 277 days and only 4 patients receiving SAT experienced infection relapse. The decision to cease SAT in 9 patients was guided by CT/PET imaging results, and all of these patients remained symptom free during follow-up. The estimated overall survival rates in this study were 78% at 1 year and 62% at 3 years. A 2023 report of 3 cases of patients with prosthetic valve infective endocarditis also found that SAT could be safely ceased when guided by PET results [50].

In terms of identifying patients at higher risk of failing SAT, it is unclear if surgical management prior to SAT alters prognosis. A study published in 2014 found that all 10 patients managed with SAT without surgical intervention did not experience reinfection during the first year of treatment, although 3 patients had infection relapse after the first year of treatment. An earlier study of patients receiving SAT but no surgical management, predominantly for VGI and prosthetic valve infection, found that 51% of participants were still receiving SAT after 1 year. In contrast, a 2015 study of patients with mycotic aneurysm who had surgical management along with SAT found that 71% of participants were still alive at 5 years. Another study found a somewhat similar 5-year survival rate of 59% ± 8%. Patients in this study also had extensive surgical management; their aortic graft enteric erosions or fistulae were repaired with rifampin-soaked grafts and omental coverage before they were commenced on SAT.

### Cardiac Implantable Electronic Device Infections

The majority of the SAT literature for CIEDIs is focused on patients with left-ventricular assist device (LVAD) infections. The organisms identified in CIEDI requiring long-term antibiotic suppression mirror those of PJI and VGI; staphylococcal species are the most common infecting organism. Interestingly, doxycycline was identified as the most common SAT agent more frequently than beta-lactam agents.

The literature on suppressive antibiotics in CIEDI focuses on patients with ventricular assist devices [48]. Most of this literature is in the form of small retrospective cohort studies. A 2020 retrospective cohort study of 24 patients examining the use of SAT for CIED driveline infections found a 50% success rate. In contrast, a 2021 retrospective study of 69 patients with superficial driveline infections, of whom 43 received SAT, found that SAT did not significantly reduce the risk of infection relapse. Two earlier studies of patients receiving SAT for LVAD-associated infections found similar failure rates of 29% and 31% after an average treatment duration of 5.8 months and 1.6 years, respectively. Interestingly, the study that found a 29% failure rate in patients receiving SAT found that only 11% of patients not receiving suppressive antibiotics experienced infection relapse. It is possible that the SAT cohort in this study had a substantially worse initial prognosis and were prescribed long-term antibiotics because they were considered less likely to be successfully treated.

In terms of survival rate, a 2017 study found that patients with CIEDI on SAT only survived for an average of 1.43 years. Patients in this study did not have their CIEDs removed. In contrast, a 2022 study found an overall 5-year survival rate of 92% for patients with LVAD infection who had received SAT until heart transplant. In this study, the patients receiving SAT for LVAD infections did not have worse post–heart transplant outcomes compared with those with resolved or no LVAD infections. This retrospective study only investigated patients who had received heart transplants, and accordingly, patients who were not candidates for transplant (due to comorbidity or low likelihood of a positive outcome) were excluded, which may explain the unusually high SAT-associated success rate compared with previous studies.

Mortality is high for patients with LVAD infection on SAT. At present, we are unable to ascertain when and if SAT can be safely stopped, especially in patients with retained devices. Device retention is associated with high mortality, but SAT may delay failure. In a highly comorbid cohort with limited life expectancy, a longer relapse-free interval is a potentially significant consideration.

Considering this evidence, we suggest that the follow-up and management of patients on SAT for CIEDIs be tailored to each individual with close monitoring of biomarkers and surveillance for relapse.

### Osteomyelitis and Spinal Hardware Infections

Apart from PJI, there is very limited evidence for the use of suppressive antibiotics in osteomyelitis. A review published in 2022 provided general recommendations for the diagnosis and treatment of acute and chronic osteomyelitis. This review suggested SAT of greater than a 3-month duration may improve treatment outcomes in patients with implant-associated osteomyelitis. In the reviewed literature, *S. aureus* was the most common infecting organism, and beta-lactams, doxycycline, and trimethoprim-sulfamethoxazole were the most used antibiotic agents for SAT.

Suppressive antibiotic therapy appears to be associated with good outcomes in early spinal implant–associated infection treated with debridement and retention. A retrospective cohort review found that SAT improved 2-year survival in patients with spinal-hardware infections treated with DAIR, compared with those who had DAIR but did not receive SAT (80% vs 33%). The authors also found that SAT reduced the risk of treatment failure in patients with infection occurring within 30 days of spinal implant, but not in late-onset infection. Similarly, a 2016 case series found that patients with early-onset multidrug-resistant spinal surgical site infections achieved clinical and biochemical resolution of infection after debridement and intravenous and long-term oral antibiotics. The patients in this series typically developed their infections within 3 weeks of their initial spinal surgery and underwent debridement within 3 days of infection diagnosis.

Longer durations of SAT do not appear to have increased survival benefit. A retrospective cohort study investigating the use of SAT in patients with a variety of orthopedic prostheses (55% of patients had spinal hardware infections) found that a 3-month duration of SAT was associated with treatment success, but a treatment duration of 6 months was not. Interestingly, a 2020 retrospective cohort study found that continuation of suppressive antibiotics beyond 12 months was inversely associated with successful treatment. Indeed, a study found that 10 of 22 patients initially treated with SAT for a median of 468 days had not experienced infection recurrence after a median follow-up of 872 days. Of the remaining 12 patients, 5 experienced failure while on treatment and were still receiving SAT at the time of last follow-up. From these studies it is possible that SAT improves patient survival in the first few months after infection, but it is unclear if continuing SAT beyond 6 months to 1 year is beneficial.

The literature on the use of suppressive antibiotics in other subtypes of osteomyelitis is even more limited than spinal-implant infections. A retrospective cohort study published in 2015 examined the use of SAT in patients with foot or long-bone osteomyelitis. This study reported that 60% of patients had successful suppression of their infections with long-term antibiotics. The duration of SAT in the study ranged from 30 to 466 days. Most infections in this study were polymicrobial, and most patients received doxycycline.

From this literature we make the following recommendation: SAT cessation may be considered in patients who have had debridement and implant retention after treatment has continued for 6 months to 1 year. As with other indications, patients should be closely followed in the first few months after SAT is stopped.

A summary of the current SAT literature is presented in Table 1.

**Table 1.** Overview of the Current Suppressive Antibiotic Therapy Literature

	Prospective Cohort Study	Case-Control Study	Retrospective Cohort Study	Case Series	Case Report
PJI	3 [17, 32]	1 [33]	18 [14, 18–25, 28, 29, 31, 35, 40]	6 [15, 16, 38, 39]	1
VGI	0	0	7	6 [41, 42, 47, 50]	7 [43–46, 49]
CIEDI	0	0	6	1	1 [48]
OM	0	0	3	2	1

Data are presented as number of studies with references in brackets.

Abbreviations: CIEDI, cardiac implantable electronic device infection; OM, osteomyelitis; PJI, prosthetic joint infection; VGI, vascular graft infection.

### Adverse Effects Associated With Suppressive Antibiotic Therapy

Cross-sectional cohort studies typically found high levels of adverse effects among the SAT patient cohort. Two recent studies investigating SAT for variable indications found adverse effect rates of 41% and 52%. The most common adverse effects were gastrointestinal disturbances and asymptomatic bacteriuria. Rates of adverse effects in retrospective studies are not always discussed in detail, and at times, a list of causative agents and a list of adverse effects are described, but the agents responsible for the particular adverse effects are not elucidated. Including the antibiotic and the adverse effect attributable to it would be beneficial for treating clinicians and patients receiving SAT.

A cross-sectional cohort study that assessed patient experiences of long-term antibiotic therapy, the majority of whom were receiving suppressive therapy, found that, although 41% experienced adverse effects, 72% of participants still reported complete adherence to their antibiotic therapy. This suggests that the fear of severe infection recurrence compels patients to continue their antibiotics despite the side effects, or that the side effects are not significantly bothersome. There are many reports of patients switching or ceasing their SAT regime due to adverse effects; however, the impact on quality of life is generally not described [15, 19–26, 28, 29].

Adverse effects associated with SAT for various indications are shown in Table 2.

**Table 2.** Adverse Effects Associated With Suppressive Antibiotic therapy

	Allergy	Cytopenia^a^	*Clostridioides difficile*	Cost/Dosing Schedule	GI Disturbance^b^	Malaise/Weariness	Neurotoxicity/Peripheral Neuropathy	Skin Disturbance^c^	Recurrent Infection	Renal Failure
Amoxicillin—clavulanic acid	…	…	2 [29]	…	1 [21]	…	…	…	…	…
Amoxicillin	1 [24]	…	…	…	1 [24]	1 [21]	…	1 [32]	…	1 [24]
Cefadroxil	…	…	…	…	…	…	…	…	1 [15]	…
Cefalexin	…	…	…	…	…	…	…	1 [15]	1 [15]	…
Dicloxacillin	…	…	…	…	2 [15, 16]	…	…	2 [15, 16]	1 [15]	…
Imipenem	…	…	…	…	…	1 [24]	…	…	…	…
Ciprofloxacin	…	1	…	1 [28]	2	…	…	…	…	…
Moxifloxacin	…	…	…	…	1 [21]	…	…	…	…	…
Ofloxacin	…	…	…	…	…	…	1 [24]	…	…	…
Clindamycin	…	…	1 [32]	…	2 [21]	1 [24]	…	…	…	…
Doxycycline	…	…	161	1 [28]	4 [24, 26, 28]	…	…	2 [24, 26]	…	…
Minocycline	…	…	…	…	1 [21]	…	…	3 [18, 21, 40]	…	…
TMP-SMX	…	1 [24]	1 [20]	…	2 [26]	…	1 [24]	2	…	3 [20, 24, 28]
Linezolid	…	2	…	…	…	…	…	1 [28]	…	1 [28]
Metronidazole	…	…	…	…	…	…	1	…	…	…
Rifampin	…	…	…	…	1	…	…	…	…	…
Pristinamycin	1 [24]	…	…	…	…	…	…	…	…	…

Data are presented as number of studies with references in brackets. Further adverse events are summarized in Supplementary Tables a and b.

Abbreviations: GI, gastrointestinal; TMP-SMX, trimethoprim-sulfamethoxazole.

^a^Cytopenia: anemia/hematotoxicity/thrombocytopenia.

^b^GI disturbance: digestive intolerance/loss of appetite/nausea/vomiting/severe nausea/inappetence/vomiting/diarrhea.

^c^Skin disturbance: phototoxicity/photosensitization/pruritus/rash/skin discoloration/hyperpigmentation/tooth discoloration.

### Antimicrobial Resistance and the Microbiome in Suppressive Antibiotic Therapy

The potential for SAT to promote the development of antibiotic resistance has been identified as a concern in the literature as early as 1998 [16]. Of the available literature, the most accurate picture of antimicrobial resistance present in the SAT population likely comes from a 2022 5-year longitudinal cohort study. This study found that 56% of SAT patients were colonized with multi-resistant bacteria, 30% of which had developed subsequent to SAT commencement. Retrospective studies across the 4 SAT indications found resistance rates between 3% and 40% [15, 19, 22, 23, 31]. Resistance against ciprofloxacin, trimethoprim-sulfamethoxazole [15], rifampicin [19], and tetracyclines has been described in various studies [22, 23].

Another potential adverse effect of SAT is disruption of the intestinal microbiome—the ecosystem of microorganisms (predominantly bacteria) that reside on and in the body surfaces. Impacts of short-term antibiotics on the gastrointestinal microbiome have been described previously, whereas the consequences of long-term suppressive antibiotics have not been researched extensively [2]. A 2020 study found that microbiome diversity was lower than expected in some participants receiving SAT, but not to a level of statistical significance. Although it was not demonstrated in this study, the possibility that SAT promotes the development or enrichment of antimicrobial resistance within an individual's microbiome is still of concern. The relevance of microbiome distortion because of SAT will be elucidated over time as the relationship between microbiome composition and health is researched more extensively. Although not common, *Clostridioides difficile* infection as a result of SAT has been reported throughout the literature [16, 29, 31, 32].

### Suggestions for Improving the Clinical Applicability of Future Suppressive Antibiotic Therapy Research

A common theme raised by many SAT review papers is that the quality of the current primary evidence makes it difficult to provide recommendations for clinical practice. Some potential strategies for future research include the following:

1. Stratifying analyses so that patients with different factors such as surgical or medical management prior to SAT are analyzed separately. Studies that examine a strictly defined demographic, such as a specific age criterion or having multiple previous prosthetic-associated surgeries, often find lower success rates [26].
2. Where possible, selecting comparison groups of patients not receiving SAT. This may provide a more realistic view of the efficacy of SAT. We acknowledge that the patients in the non-SAT group are potentially not fully comparable to those receiving SAT—if they were as comorbid as the SAT cohort, they presumably would have been prescribed SAT as well.
3. Designing studies with larger cohort sizes to increase the statistical power of the results. Studies that have achieved higher cohort sizes are generally multicentered or use nationally available databases to identify patients [24, 29–31].
4. Including patients who did not receive a minimum duration of SAT, usually due to death from their infection or another cause as a separate early failure group. The practice of excluding these patients entirely likely overestimates the success rate of SAT by constructing a more resilient cohort with a better initial prognosis for the analysis.

Due to the lack of clarity in the suppressive antibiotic literature, clinicians and patients may err on the side of caution and continue SAT indefinitely, exposing both the patient and the community to the known and potential risks of this therapy.

## GENERAL RECOMMENDATIONS FOR PRACTICE REGARDING SUPPRESSIVE ANTIBIOTIC THERAPY

### Selecting Patients for Suppressive Antibiotic Therapy

There are 2 broad categories of patients who may be started on SAT. The first is in patients who have failed primary treatment of their infection. Failure is characterized by persistence of clinical infection or infectious biomarkers after the indicated surgery and intravenous antibiotic course.

The second group is patients who are not candidates for the indicated surgical (with intravenous antibiotic) route. These patients are typically frail, highly comorbid, with limited life expectancy. In these patients, the role of suppressive antibiotic therapy is weighed against the risk of aggressive surgical interventions, to provide more satisfactory patient outcomes.

The transition from a prolonged oral curative course to one with suppressive intent is not always clear; in our previous studies we have considered oral courses initially prescribed with an intended duration over 1 year to be suppressive therapy.

### Monitoring Patients on Suppressive Antibiotic Therapy

We recommend monitoring patients receiving SAT more frequently in the initial year after commencing therapy, and then every 6 months or annually once the patient's clinical and biochemical signs of infection have normalized. Evidence suggests that patients are more likely to fail SAT early in the treatment course; thus, more frequent monitoring is warranted over this period [19, 29].

### Duration of Suppressive Antibiotic Therapy

Some authors have demonstrated that continuing SAT beyond 1 year does not continue to reduce the risk of treatment failure. In our experience, cessation of SAT can be considered if the following criteria are met. The patient should have stable inflammatory markers (eg, white blood cell count, ESR, and CRP) within normal reference ranges for at least 1 year, without clinical signs of infection such as ongoing pain or discharge from a prosthetic joint site. If appropriate baseline imaging exists, the infected site should be re-imaged to compare disease activity. Finally, patient preference should be at the forefront of the decision to cease SAT. Patient factors to consider are how well the patient has tolerated SAT, what is the patient's life expectancy, and how devastating infection recurrence would be for that patient. In frailer patients, with limited life expectancy in whom an infection recurrence may prove fatal, it may be reasonable to continue SAT indefinitely.

## CONCLUSIONS

Currently, a wide range of terminology is used, including “long-term suppressive therapy,” “indefinite chronic suppression,” and “indefinite oral antimicrobial suppression.” We recommend “suppressive antibiotic therapy” as a standardized term that accurately and succinctly describes the practice of using long courses of antibiotics to indefinitely suppress bacterial infection, so that relevant data are not unintentionally excluded from systematic reviews. As antimicrobial resistance and the levels of comorbidity in the population increase worldwide, the number of patients prescribed SAT will likely increase. Therefore, it is important that both patients and clinicians have accurate information about the treatment strategy to promote safe patient-centered care.

## Supplementary Data

Supplementary materials are available at *Clinical Infectious Diseases* online. Consisting of data provided by the authors to benefit the reader, the posted materials are not copyedited and are the sole responsibility of the authors, so questions or comments should be addressed to the corresponding author.

## Supplementary Material

ciae302_Supplementary_Data

## Notes

***Financial support*.** The authors received no external funding for the research, synthesis, and writing of this paper.
