# Supplementary material for: The Use of Long-term Antibiotics for Suppression of Bacterial Infections
Source: Clin Infect Dis. 2024 Jun 4;79(4):848–54. doi: 10.1093/cid/ciae302 (PMC11478772; doi:10.1093/cid/ciae302)
Supplement: ciae302_Supplementary_Data [file ciae302_supplementary_data.zip › CID references 51-90.docx]

References 51 - 90

51. Sanders PTJ, Bus MPA, Scheper H, van der Wal RJP, van de Sande MAJ, Bramer JAM, et al. Multiflora and Gram-Negative Microorganisms Predominate in Infections Affecting Pelvic Endoprostheses Following Tumor Resection. J Bone Joint Surg Am. 2019;101(9):797-803.

52. Vallejo Camazon N, Mateu L, Cediel G, Escolà-Vergé L, Fernández-Hidalgo N, Gurgui Ferrer M, et al. Long-term antibiotic therapy in patients with surgery-indicated not undergoing surgery infective endocarditis. Cardiol J. 2021;28(4):566-78.

53. Erb S, Sidler JA, Elzi L, Gurke L, Battegay M, Widmer AF, et al. Surgical and Antimicrobial Treatment of Prosthetic Vascular Graft Infections at Different Surgical Sites: A Retrospective Study of Treatment Outcomes. PLoS ONE. 2014;9(11):e112947.

54. Baddour LM, Infectious Diseases Society of America's Emerging Infections N. Long-term suppressive antimicrobial therapy for intravascular device-related infections. The American journal of the medical sciences. 2001;322(4):209-12.

55. Lau C, Gaudino M, de Biasi AR, Munjal M, Girardi LN. Outcomes of Open Repair of Mycotic Descending Thoracic and Thoracoabdominal Aortic Aneurysms. Ann Thorac Surg. 2015;100(5):1712-7.

56. Oderich GS, Bower TC, Hofer J, Kalra M, Duncan AA, Wilson JW, et al. In situ rifampin-soaked grafts with omental coverage and antibiotic suppression are durable with low reinfection rates in patients with aortic graft enteric erosion or fistula. J Vasc Surg. 2011;53(1):99-106, 7.e1-7; discussion -7.

57. Coselli JS, Crawford ES, Williams TW, Jr., Bradshaw MW, Wiemer DR, Harris RL, et al. Treatment of postoperative infection of ascending aorta and transverse aortic arch, including use of viable omentum and muscle flaps. Ann Thorac Surg. 1990;50(6):868-81.

58. Saleem BR, Meerwaldt R, Tielliu IF, Verhoeven EL, van den Dungen JJ, Zeebregts CJ. Conservative treatment of vascular prosthetic graft infection is associated with high mortality. Am J Surg. 2010;200(1):47-52.

59. Nienaber JJ, Kusne S, Riaz T, Walker RC, Baddour LM, Wright AJ, et al. Clinical manifestations and management of left ventricular assist device-associated infections. Clin Infect Dis. 2013;57(10):1438-48.

60. Riaz T, Nienaber JJ, Baddour LM, Walker RC, Park SJ, Sohail MR. Cardiovascular implantable electronic device infections in left ventricular assist device recipients. Pacing Clin Electrophysiol. 2014;37(2):225-30.

61. Jennings DL, Chopra A, Chambers R, Morgan JA. Clinical outcomes associated with chronic antimicrobial suppression therapy in patients with continuous-flow left ventricular assist devices. Artif Organs. 2014;38(10):875-9.

62. Hamad Y, Blanco‐Guzman MO, Olsen MA, Wang X, Vader J, Verma A, et al. The role of chronic suppressive antibiotics therapy in superficial drive line infection relapse of left ventricular assist devices: A retrospective cohort from a tertiary care center. Transplant infectious disease. 2021;23(4):e13686-n/a.

63. Lambadaris M, Vishram-Nielsen JKK, Amadio JM, Husain S, Rao V, Billia F, et al. Association between continuous-flow left ventricular assist device infections requiring long-term antibiotic use and post-heart transplant morbidity and mortality. J Card Surg. 2022;37(1):96-104.

64. Tan EM, DeSimone DC, Sohail MR, Baddour LM, Wilson WR, Steckelberg JM, et al. Outcomes in Patients With Cardiovascular Implantable Electronic Device Infection Managed With Chronic Antibiotic Suppression. Clin Infect Dis. 2017;64(11):1516-21.

65. Radcliffe C, Doilicho N, Niu YS, Grant M. Efficacy and safety of chronic antimicrobial suppression therapy for left ventricular assist device driveline infections: A single-center descriptive experience. Transpl Infect Dis. 2020;22(5):e13379.

66. Phadke VK, Pouch SM. Contemporary Management Strategies in VAD Infection. Current heart failure reports. 2020;17(4):85-96.

67. Joost I, Bothe W, Pausch C, Kaasch A, Lange B, Peyerl-Hoffmann G, et al. Staphylococcus aureus bloodstream infection in patients with ventricular assist devices-Management and outcome in a prospective bicenter cohort. J Infect. 2018;77(1):30-7.

68. Masters EA, Ricciardi BF, Bentley KLdM, Moriarty TF, Schwarz EM, Muthukrishnan G. Skeletal infections: microbial pathogenesis, immunity and clinical management. Nature Reviews Microbiology. 2022;20(7):385-400.

69. Beydoun N, Tandon S, Krengel S, Johnson E, Palacio Bedoya F, Moore M, et al. A Retrospective Chart Review on the Role of Suppressive Therapy in the Management of Spinal Infections Involving Hardware. Open Forum Infect Dis. 2020;7(7):ofaa253.

70. Keller SC, Cosgrove SE, Higgins Y, Piggott DA, Osgood G, Auwaerter PG. Role of Suppressive Oral Antibiotics in Orthopedic Hardware Infections for Those Not Undergoing Two-Stage Replacement Surgery. Open Forum Infect Dis. 2016;3(4):ofw176.

71. Kowalski TJ, Berbari EF, Huddleston PM, Steckelberg JM, Mandrekar JN, Osmon DR. The management and outcome of spinal implant infections: contemporary retrospective cohort study. Clin Infect Dis. 2007;44(7):913-20.

72. Nowak MA, Winner JS, Beilke MA. Prolonged oral antibiotic suppression in osteomyelitis and associated outcomes in a Veterans population. Am J Health Syst Pharm. 2015;72(23 Suppl 3):S150-5.

73. Miyazaki S, Kakutani K, Maeno K, Takada T, Yurube T, Kurosaka M, et al. Surgical debridement with retention of spinal instrumentation and long-term antimicrobial therapy for multidrug-resistant surgical site infections after spinal surgery: a case series. Int Orthop. 2016;40(6):1171-7.

74. Ferry T, Conrad A, Senneville E, Roux S, Dupieux-Chabert C, Dinh A, et al. Safety of Tedizolid as Suppressive Antimicrobial Therapy for Patients With Complex Implant-Associated Bone and Joint Infection due to Multidrug-Resistant Gram-Positive Pathogens: Results From the TediSAT Cohort Study. Open Forum Infectious Diseases. 2021;8(7):ofab351.

75. Prendki V, Sergent P, Barrelet A, Oziol E, Beretti E, Berlioz-Thibal M, et al. Efficacy of indefinite chronic oral antimicrobial suppression for prosthetic joint infection in the elderly: a comparative study. International journal of infectious diseases : IJID : official publication of the International Society for Infectious Diseases. 2017;60:57-60.

76. Barry JJ, Geary MB, Riesgo AM, Odum SM, Fehring TK, Springer BD. Irrigation and Debridement with Chronic Antibiotic Suppression Is as Effective as 2-Stage Exchange in Revision Total Knee Arthroplasty with Extensive Instrumentation. J Bone Joint Surg Am. 2021;103(1):53-63.

77. Valencia JCB, Abdel MP, Virk A, Osmon DR, Razonable RR. Destination Joint Spacers, Reinfection, and Antimicrobial Suppression. Clin Infect Dis. 2019;69(6):1056-9.

78. Koeppe J, Johnson S, Morroni J, Siracusa-Rick C, Armon C. Suppressive Antibiotic Therapy for Retained Infected Prosthetic Joints: Case Series and Review of the Literature. Infectious diseases in clinical practice (Baltimore, Md). 2008;16(4):224-9.

79. Everts RJ, Chambers ST, Murdoch DR, Rothwell AG, McKie J. Successful antimicrobial therapy and implant retention for streptococcal infection of prosthetic joints. ANZ J Surg. 2004;74(4):210-4.

80. Ferry T, Batailler C, Conrad A, Triffault-Fillit C, Laurent F, Valour F, et al. Correction of Linezolid-Induced Myelotoxicity After Switch to Tedizolid in a Patient Requiring Suppressive Antimicrobial Therapy for Multidrug-Resistant Staphylococcus epidermidis Prosthetic-Joint Infection. Open Forum Infectious Diseases. 2018;5(10):ofy246.

81. Simmons CD, Ali AT, Foteh K, Abate MR, Smeds MR, Spencer HJ, et al. Unilateral inline replacement of infected aortofemoral graft limb with femoral vein. J Vasc Surg. 2017;65(4):1121-9.

82. Morris GE, Friend PJ, Vassallo DJ, Farrington M, Leapman S, Quick CR. Antibiotic irrigation and conservative surgery for major aortic graft infection. J Vasc Surg. 1994;20(1):88-95.

83. Pencavel TD, Singh-Ranger G, Crinnion JN. Conservative treatment of an early aortic graft infection due to Acinetobacter baumanii. Annals of vascular surgery. 2006;20(3):415-7.

84. Lauk O, Fulchini R, Hasse BK, Schmitt-Opitz I. Aortobronchial fistula and Listeria endograft infection after repeated T/EVAR: a rare combination. BMJ Case Reports. 2020;13(3):e229924.

85. Itoh N, Akazawa N, Ishibana Y, Hamada S, Hagiwara S, Murakami H. Femoral osteomyelitis caused by oral anaerobic bacteria with mixed bacteremia of Campylobacter rectus and Parvimonas micra in a chronic periodontitis patient: a case report. BMC Infect Dis. 2022;22(1):613.

86. Lau JSY, Bhatt S, Streitberg R, Bryant M, Korman TM, Woolley I. Surveillance of life-long antibiotics-A cross-sectional cohort study assessing patient attitudes and understanding of long-term antibiotic consumption. Infect Dis Health. 2019;24(4):179-86.

87. Escudero-Sanchez R, Ponce-Alonso M, Barragan-Prada H, Morosini MI, Canton R, Cobo J, et al. Long-Term Impact of Suppressive Antibiotic Therapy on Intestinal Microbiota. Genes. 2020;12(1).

88. Kiss C, Connoley D, Connelly K, Horne K, Korman T, Woolley I, et al. Long-Term Outcomes in Patients on Life-Long Antibiotics: A Five-Year Cohort Study. Antibiotics (Basel). 2022;11(1).

89. Maze MJ, Laws P, Buckenham T, Pithie A, Gallagher K, Metcalf S, et al. Outcomes of Infected Abdominal Aortic Grafts Managed with Antimicrobial Therapy and Graft Retention in an Unselected Cohort. European Journal of Vascular and Endovascular Surgery. 2013;45(4):373-80.

90. MacPhail A, Korman T, Woolley I, Lau J. Long term antibiotic prescribing in the community: 6 years of Australian national data. Journal of the American Pharmacists Association.
