# Supplementary material for: The Use of Long-term Antibiotics for Suppression of Bacterial Infections
Source: Clin Infect Dis. 2024 Jun 4;79(4):848–54. doi: 10.1093/cid/ciae302 (PMC11478772; doi:10.1093/cid/ciae302)
Supplement: ciae302_Supplementary_Data [file ciae302_supplementary_data.zip › Supplementary material- tables.docx]

Supplementary table a: Adverse effects associated with suppressive antibiotic combinations

|  | Allergy | C difficile | GI disturbance | Drug interaction | Dry mouth | Malaise / weariness | Renal failure |
| --- | --- | --- | --- | --- | --- | --- | --- |
| Doxycycline + ciprofloxacin |  |  | 1^65^ |  |  |  |  |
| Doxycycline + rifampin |  |  | 2^28, 43^ |  | 1^26^ | 1^24^ |  |
| Minocycline + rifampin |  |  | 1^16^ |  |  |  |  |
| Amoxicillin + levofloxacin |  |  |  |  |  |  | 1^24^ |
| Clindamycin + fusidic acid |  | 1^24^ |  | 1^24^ |  | 1^24^ |  |
| Levofloxacin + rifampin |  |  | 1^24^ |  |  |  |  |
| Ofloxacin + rifampin |  |  | 1^24^ |  |  | 1^24^ | 1^24^ |
| Ofloxacin + clindamycin | 1^24^ |  |  |  |  |  |  |
| Oxacillin + rifampin |  |  |  |  |  | 1^24^ |  |
| Pristinamycin + rifampin |  |  | 1^24^ |  |  |  | 1^24^ |
| Teicoplanin + rifampin |  |  |  |  |  |  | 1^24^ |
| TMP-SMX + fusidic acid | 1^24^ |  |  |  |  |  |  |
| TMP-SMX + rifampin |  |  |  |  |  | 1^24^ |  |

GI disturbance: Digestive intolerance/ loss of appetite/ nausea/vomiting / severe nausea / inappetence / vomiting / diarrhoea

Supplementary table b: Other miscellaneous adverse effects associated with suppressive antibiotics

| Adverse effects | Antibiotic |
| --- | --- |
| Achilles tendinopathy | Ciprofloxacin^19^ |
| Antibiotic resistance | TMP-SMX^15^ |
| Dizziness/vertigo | Minocycline^21^ |
| Drug interaction | Rifampin^28^ |
| Dry mouth | Amoxicillin^21^ |
| LFT derangement | Clindamycin^47^ |
| Pleural effusion | TMP-SMX^26^ |
| Raised INR | TMP-SMX^65^ |
